# Comparative study of encoded and alignment-based methods for virus taxonomy classification

**DOI:** 10.1038/s41598-023-45461-0

**Published:** 2023-10-31

**Authors:** Muhammad Arslan Shaukat, Thanh Thi Nguyen, Edbert B. Hsu, Samuel Yang, Asim Bhatti

**Affiliations:** 1 Institute for Intelligent Systems Research and Innovation (IISRI), Deakin University, Victoria, Australia; 2 Faculty of Information Technology, Monash University, Victoria, Australia; 3 Department of Emergency Medicine, Johns Hopkins University, Maryland, USA; 4 Department of Emergency Medicine, Stanford University, California, USA

**Keywords:** Computational biology and bioinformatics, Classification and taxonomy, Computational models, Phylogeny

## Abstract

The emergence of viruses and their variants has made virus taxonomy more important than ever before in controlling the spread of diseases. The creation of efficient treatments and cures that target particular virus properties can be aided by understanding virus taxonomy. Alignment-based methods are commonly used for this task, but are computationally expensive and time-consuming, especially when dealing with large datasets or when detecting new virus variants is time sensitive. An alternative approach, the encoded method, has been developed that does not require prior sequence alignment and provides faster results. However, each encoded method has its own claimed accuracy. Therefore, careful evaluation and comparison of the performance of different encoded methods are essential to identify the most accurate and reliable approach for virus taxonomy classification. This study aims to address this issue by providing a comprehensive and comparative analysis of the potential of encoded methods for virus classification and phylogenetics. We compared the vectors generated for each encoded method using distance metrics to determine their similarity to alignment-based methods. The results and their validation show that K-merNV followed by CgrDft encoded methods, perform similarly to state-of-the-art multi-sequence alignment methods. This is the first study to incorporate and compare encoded methods that will facilitate future research in making more informed decisions regarding selection of a suitable method for virus taxonomy.

## Introduction

COVID-19 infection primarily spreads through respiratory droplets and can cause symptoms ranging from mild such as fever and cough, to severe such as difficulty in breathing and pneumonia^1^. The global impact of COVID-19 cannot be overstated and continues to evolve as new virus variants emerge. Understanding virus taxonomy can aid in virus management by allowing researchers and healthcare providers to identify and track various types of viruses. For example, if a new virus is discovered, establishing its taxonomy might help scientists understand its traits and how it may behave.

Virus taxonomy can support the development of vaccines and treatments. Researchers can assess which types of vaccinations or therapies would be effective by identifying the family and species of a virus. This knowledge may potentially be used to direct the creation of novel therapies that focus on particular viral traits. By using computational techniques like alignment-based methods, it is possible to classify viruses according to their genomic sequences and deduce their evolutionary relationships.

Alignment-based methods for classifying genes rely on finding optimal alignments between sequences using scoring systems. They are often performed using software such as ClustalW or MUSCLE, which can align the sequences and calculate a score that reflects their similarity^2,3^. Once the sequences are aligned, a phylogenetic tree^4^ can be constructed using various algorithms, such as the neighbor-joining method^5^ or the maximum likelihood method^6^. The resulting tree reflects the evolutionary relationships between the organisms based on their genomic sequences. While accurate, these techniques are computationally expensive, which makes them unsuitable for assessing huge datasets^7^. To overcome these limitations, a variety of alignment-free techniques have been developed in the signal processing domain over the last two decades with promising performance^8^.

Alignment-free methods do not employ sequence alignments to compare and classify sequences; instead, they extract features or patterns and use them to compare and classify sequences. Because of their efficiency, scalability, and ability to handle big datasets, alignment-free approaches have grown in popularity^9–11^.

There are several alignment-free (i.e., encoded) approaches available^7,11–14^, each with its own stated accuracy. This study comprehensively reviews encoded methods that employ various approaches. We aim to investigate the behaviour of these encoded methods and determine how they address the uncertainty associated with generating reliable results. By identifying dependable and rapid techniques, we seek to highlight promising avenues for future research. Our work offers several important contributions to the field, including:

- Providing a first of its kind, comprehensive and comparative review of seventeen different encoded methods for ten different data-sets of varying lengths.
- Comparing the effectiveness of encoded methods with the well-established non-encoded methods. By doing so, we have identified the strengths and weaknesses of encoded methods and provided insights into their respective performances.
- Identifying the most reliable and fast encoded method, which could be used as an alternative to the computationally expensive alignment method.
- Publishing the datasets and codes used in this study online, to enable other researchers to replicate and build upon the findings of the study.
- Offering supplementary documents that provide phylogenetic trees and metrics results for each method and dataset. This information will be useful to researchers who want to delve further into the data to gain a more detailed understanding of the results.

## Materials and methods

This study investigated a total of twenty different methods: four non-encoded multi-aligned methods and seventeen encoded methods. We employed ten different datasets using three separate software tools - Matlab, MEGA, and NGphylogeny (online). The similarity of encoded methods is compared to four state-of-the-art multi-sequence alignment methods. Two of these methods, ClustalW^3^ and MUSCLE^15^, were implemented on the software package MEGA 11^16^. The other two methods, MAFFT^17^ and ClustalOmega^18^, were implemented on an online tool called NGphylogeny^19^.

**Figure 1:**
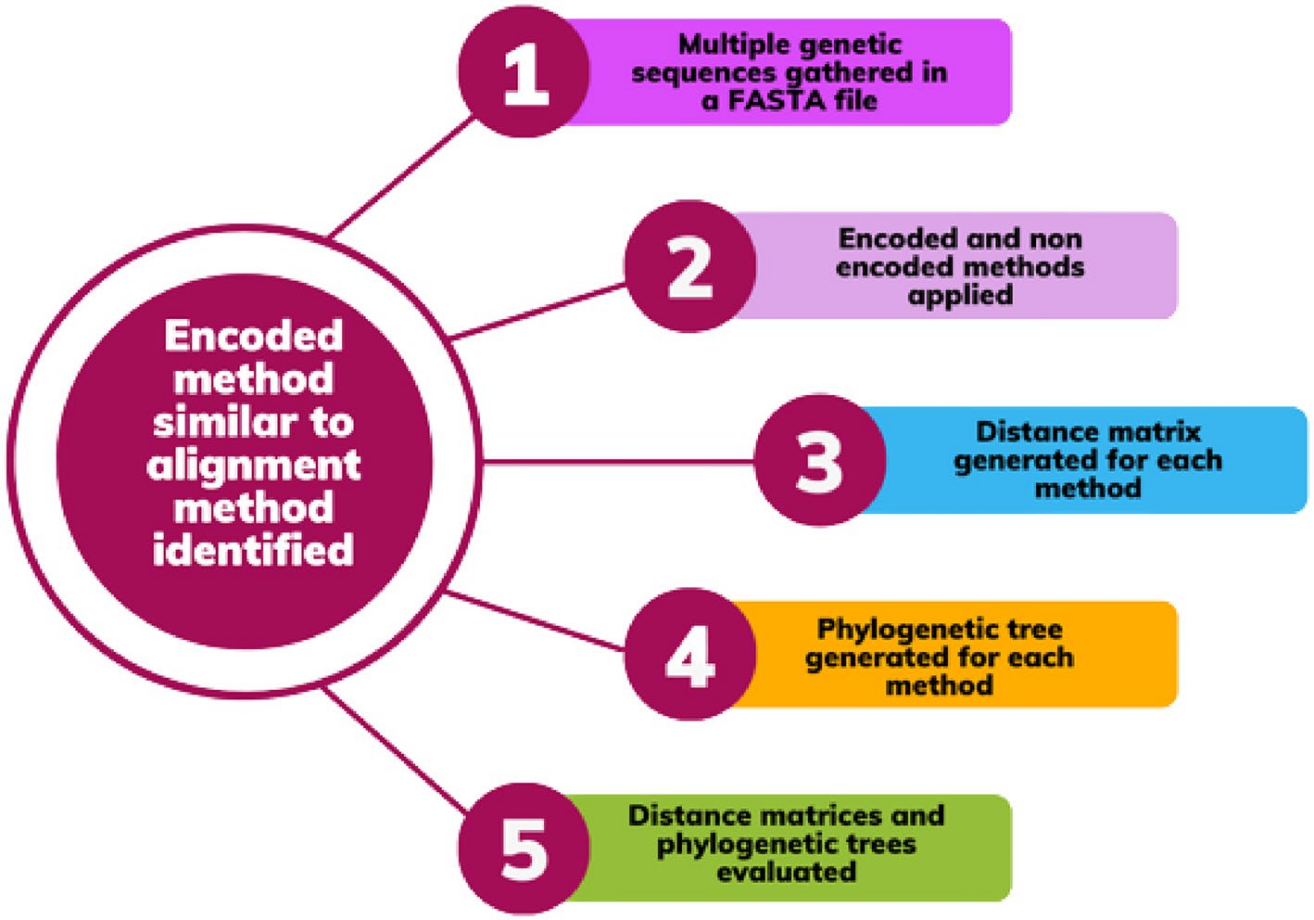
Overview of the methodology used to compare encoded and multi-aligned (non-encoded methods). The process involved generating distance matrices, comparing them to rank the encoded methods, filtering out non-similar methods, and analysing phylogenetic trees to validate the results.

This comparison allowed us to rank the encoded methods based on their similarity to the alignment method. It is worth noting that the choice of sequence alignment method may affect the comparison results. However, there are commonly used methods for multiple sequence alignment that are well-respected in the field. Additionally, use of two different software tools (MEGA11 and NGphylogeny) help to ensure that the results are robust and not influenced by a specific software implementation.

We used a funnel approach to evaluate each non-encoded method (Fig. 1). First, we generated distance matrices to record the distances between sequences for each dataset using both encoded and non-encoded methods. In order to generate an evolutionary distance matrix for a non-encoded method, it is essential to first align all the sequences in a given dataset. The Jukes-Cantor model^20^ was used to construct the matrix for each alignment (i.e., non-encoded) method to ensure that the results obtained from these methods were comparable. When comparing different encoding methods, one potential issue is that some methods may produce matrices with large values. In contrast, others may produce matrices with small values. We have normalized our matrix over a range of 0 and 1 where the minimum value is set to 0 and the maximum value is set to 1 since it is likely that the absolute value of the matrix does not reflect the differences between methods . By doing this, the matrix values are transformed into a consistent scale, which allows for fair and unbiased comparisons between different methods.

Next, we compare the distance matrix generated by each encoded method with the distance matrix generated by each non-encoded method using Euclidean distance. The method with the smallest Euclidean distance to the non-encoded method will considered to be the most similar. This comparison allowed us to rank the encoded methods based on their similarity to the alignment method. Finally, in order to validate the results obtained in the previous step, we utilised distance metrics at an arbitrary non-binary phylogenetic tree level. We then excluded any encoded methods that failed to meet the similarity criteria. To provide additional validation, we conducted a visual comparison of the phylogenetic trees. This enabled us to determine which encoded method had the least amount of difference from the multi-sequence alignment method. Through these steps, we were able to determine the effectiveness and accuracy of each encoded method and identify the most similar method to the non-encoded one.

### Dataset

In this study, datasets were incorporated from previous studies^12,14,21^, where virus genomes were collected from sources such as GenBank^22^ and GISAID^23^. To prevent any potential biases from the datasets used in previous studies, a new dataset, Dataset0 is also included in this analysis. Dataset0 has not been previously used in any encoding techniques, ensuring a fair comparison between the different encoding methods being tested. The Table 1 shows the datasets used in the study.

**Table 1:** Datasets from previous research are incorporated to evaluate the effectiveness of different encoding methods and their response to various parameters, such as the number of sequences and the maximum sequence length. To prevent any potential biases from the datasets used in previous studies, a new dataset, Dataset0 is also included in this analysis. Dataset0 has not been previously used in any encoding techniques, ensuring a fair comparison between the different encoding methods being tested.

Name	Description	Total Seqs	Min. length	Max. length
DataSet0	Viruses in the genus AlphaCoV and BetaCoV of coronaviruses, along with their subgenera in BetaCov	59	27165	31526
DataSet1	Viruses from the family Coronaviridae to classify SARS-CoV-2	56	25425	31686
DataSet2	Viruses in the genus BetaCoV to classify SARS-CoV-2 at the genus level	50	29037	31491
DataSet3	Closely related coronaviruses from the seafood market	69	27213	30311
DataSet4	Transmission modes of human coronaviruses originating from animals	106	26883	31473
DataSet5	Virus genomes obtained from human SARS-CoV-2 viruses	141	29674	29882
DataSet6	Genus within the Coronaviridae family, known to induce a range of severe diseases in the respiratory and gastrointestinal systems	34	9646	31357
DataSet7	Influenza A viruses, which are single-stranded, segmented RNA viruses categorized according to their hemagglutinin and neuraminidase viral surface proteins	38	1350	1467
DataSet8	Human rhinoviruses, which is the most common cause of upper respiratory tract	116	6944	7458
DataSet9	HPV (Human Papillomavirus) is a common sexually transmitted DNA virus responsible for cervical cancer and genital warts	400	7814	10424

### Multi aligned (non-encoded) method

#### ClustalW

ClustalW uses a progressive alignment to align protein or nucleotide sequences. It involves constructing a guide tree based on pairwise distances between the sequences and then aligning the sequences based on the order of the tree.

#### ClustalOmega

Clustal Omega uses a combination of progressive and iterative methods to align protein or nucleotide sequences. It constructs a guide tree based on pairwise distances between sequences and then aligns the sequences based on the order of the tree. It then iteratively refines the alignment to improve its accuracy.

#### MUSCLE

MUSCLE (Multiple Sequence Comparison by Log-Expectation) constructs an initial alignment of the most similar sequences and then iteratively refines the alignment to incorporate additional sequences. It uses a progressive alignment method, which starts with a rough alignment of the most similar sequences and then adds in the remaining sequences one by one.

#### MAFFT

MAFFT (Multiple Alignment using Fast Fourier Transform) uses a variety of algorithms to align sequences, including progressive pairwise alignment, iterative refinement, and consistency-based alignment. It also employs a fast Fourier transform algorithm to improve the accuracy of the alignment.

### Encoded methods

#### Atomic number

The atomic number method of gene encoding assigns each nucleotide base in DNA a corresponding atomic number. The nucleotide bases are represented by A = 70, T = 66, C = 58, and G = 78, which correspond to the number of protons in the nucleus of an atom^24^. These numerical representations offer the ability to perform counting the occurrences of specific sequences within a larger sequence and comparing the similarity between two sequences^25^. This approach has been applied in the analysis of Rubisco protein genes, where a direct mapping using atomic numbers was employed to calculate sequence fluctuation^26^.

#### Electron-ion interaction pseudopotential

The Electron-Ion Interaction Pseudopotential (EIIP) technique is a computational physics method employed to investigate the interactions between electrons and ions in materials. In this technique, specific values (C=0.1340, T=0.1335, A=0.1260, and G=0.0806) represent the relative frequency of the four nucleotides (C, T, A, and G) within a genetic sequence^27^. These values characterize the distribution of energies associated with the pseudopotentials of free electrons across the DNA sequence. The utilization of EIIP values has found practical applications in various domains, including neural networks, wavelet transform, and graph signal processing (GSP)^28^. By reflecting the pseudopotential feature of nucleotide sequences, EIIP values have proven to be valuable in fields such as bioinformatics, genomics, and molecular biology^29^.

#### Molecular mass representation

Molecular mass quantifies the total mass of all atoms present in a molecule. To represent molecular mass numerically, the individual atoms within the molecule are assigned values based on their atomic masses, typically expressed in atomic mass units (amu). In the context of gene sequence encoding, each nucleotide (C, T, A, and G) is assigned a specific numerical code, 110, 125, 134, and 150, respectively^30^^31^. By employing these encoded nucleotide sequences, various mathematical techniques, such as clustering algorithms, can be applied to analyze and identify patterns or relationships between the sequences.

#### Frequency-of-occurrence

The fractional occurrence of nucleotides and their frequencies are key parameters used in various bioinformatics analyses of DNA sequences. It can be statistically calculated based on the frequencies of their occurrence in specific regions of the genome, such as exons and introns^32^. The encoding representation of each of the four nucleotides is cytosine = 0.27215, thymine = 0.20576, adenine = 0.24300, and guanine = 0.27909^33^.

#### Pulse amplitude modulation

Pulse Amplitude Modulation (PAM) is a computational method employed in genomics to compare genomic sequence similarity. It is used to compare two genome sequences and quantify their differences in mutations. The specific real numbers assigned to denote the bases, A = -1.5, G = -0.5, T = 1.5, and C = 0.5, are arbitrary and were chosen to represent the differences between the bases in a way that is easily computable^34^. The real numbers used in the PAM scheme are not meant to represent the biological or chemical properties of the bases. Instead, they are meant to provide a convenient way to quantify differences between genomes. PAM has been utilized in comparative genomics, particularly in the analysis of native and synthetic enzymes^35^. It provides a quick and effective way to compare genomes, determine relatedness, and track genome evolution.

#### Fourier power spectrum

The Fourier power spectrum, represents the power or energy distribution of a signal in the frequency domain. The periodic patterns or repeated motifs that are present in the sequence can be identified using the Fourier Power Spectrum. Due to a variety of aspects, including repeating elements, coding regions, or structural characteristics, DNA sequences can display specific patterns or periodicities. This is achieved by breaking down the sequences into overlapping substrings and calculating the Fourier spectrum for each base^7^.

#### Chaos game representation with discrete Fourier transform

Chaos Game Representation (CGR) is a graphical representation method used in genomics to visualize the structure of genomic sequences proposed by Jeffery^36^. The mathematical equations for Chaos Game Representation of a DNA sequence can be defined as follows:

1 $$\begin{aligned} Xo = (1/2,1/2), Xn=1/2 (Xn-1+W), \end{aligned}$$

where W is the coordinates of the corner of the unit square.

In the CGR method, a DNA sequence is divided into overlapping triplets of nucleotides (codons), and the positions of the codons are plotted on a two-dimensional plane according to their corresponding amino acids. This allows the DNA sequence structure to be visualized as a pattern on the plane. Regions of the sequence that code for similar amino acids appear as clusters.

The Discrete Fourier Transform (DFT) transforms signals from the time domain to the frequency domain and represents them as a sum of sinusoids of different frequencies. The DFT provides the coefficients of these sinusoids. In genome encoding, DFT can be employed to analyze and compare DNA sequences by transforming them from their original form into a frequency-based representation^37^. The DFT coefficients provide information about the distribution of different frequencies in the sequence. They can be used to generate a spectrogram, which visualizes DNA sequence frequencies.

DFT can also be used to detect motifs and patterns in DNA sequences by looking for peaks in the spectrogram that correspond to specific frequencies. This can provide insights into the underlying structure and functional regions of DNA sequences^38^. CgrDft is a hybrid method that uses Chaos Game Representation with Discrete Fourier Transform (CgrDft) to visualize DNA sequence structure by mapping them onto a two-dimensional plane^12^.

#### Dinucleotide

Dinucleotide DNA encoding is a method of representing DNA sequences using a two-dimensional plot. In this method, sixteen different dinucleotides (two nucleotide pairs) are mapped to a unit circle, with each dinucleotide represented by a distinct position on the circle^39^. The sixteen dinucleotides are AA, AC, AG, AT, CA, CC, CG, CT, GA, GC, GG, GT, TA, TC, TG, and TT. Once the dinucleotides are mapped to the unit circle, neighboring nucleotides in the DNA sequence are encoded as points on a two-dimensional plot. It can also be used for machine learning applications such as predicting genomic features or classifying DNA sequences.

#### I Ching representation

In gene sequence encoding, the 64 codons that make up the genetic code can be expressed through binary codes. To map these binary codes to the 64 codons, one approach is to use the I Ching^40^. Each hexagram in the I Ching can be assigned its own four-digit binary code based on the arrangement of solid and broken lines. These binary codes represent the 64 codons. Codons are mapped to hexagrams whose binary codes correspond to their own binary representations. For example, the codon AUG, which codes for the amino acid methionine, can be expressed as 0100 in binary. This binary code corresponds to hexagram 23 in the I Ching composed of two solid lines at the bottom and third positions and four broken lines in the middle. Therefore, in this encoding scheme, AUG would be represented as hexagram 23.

#### Integer number

Integer number encoding is a method of representing data using integers instead of characters or symbols. In the context of DNA sequence encoding, integer number encoding involves mapping each nucleotide (C, T, A, or G) to a corresponding integer value (0, 1, 2, or 3)^41,42^. Integer number encoding can be useful in DNA sequence analysis, as it allows for efficient storage and manipulation of large datasets. It also enables the use of mathematical operations and algorithms designed for working with integers^43^. However, it is imperative to use a consistent encoding scheme to ensure that different systems and programs can interpret the data correctly^44^.

#### Inter-nucleotide distance

In the inter-nucleotide encoding scheme the sequence is represented as a series of inter-nucleotide sequences rather than as a series of nucleotides themselves^45^. One way to encode the distance between pairs of nucleotides that are a fixed length apart in the sequence is with a fixed distance of k^46^ i.e., i+k, i+2k,.., i+nk can be used to represent between nucelotides and S(i), S(i+k), can be encodes as k, k1 if the same nucelotides occur at each position. Inter-nucleotide distance encoding can be useful in some applications^47^, as it can reduce the dimensionality of the data and facilitate certain types of analysis. However, it may not be appropriate for all types of gene sequence analysis, as it discards some information about the specific nucleotides in the sequence^48^.

#### Minimum entropy

Minimum entropy refers to the minimum amount of information needed to represent the sequence or the level of compression achieved for the sequence^49^. The entropy of a gene sequence can be calculated using the formula:

2 $$\begin{aligned} (H(M) = - \sum p(x) log p(x)), \end{aligned}$$

where p(x) is the probability of each nucleotide base in the sequence. It also measures the average number of bits required to represent each distance and can be used to compress the gene sequence in a way that preserves the order and relative distances of the nucleotides.

#### Thermodynamic encoding

Thermodynamic encoding of gene sequences is a method of encoding gene sequences based on enthalpy values. The idea is to use the thermodynamic stability of DNA’s double helix structure to encode sequence information in a way that is more robust to errors and noise^50^. The DNA double helix structure is stable due to interactions between nucleotide bases. The base pairs A-T and G-C form hydrogen bonds that stabilize the double helix structure^51^. The enthalpy change of hydrogen bonding interactions between DNA strands can be calculated using thermodynamic principles. The enthalpy values of these interactions can be used to encode sequence information in a way that is more resistant to errors and mutations.

#### K-mer encoding

K-mer encoding simplifies long DNA sequences into smaller chunks. For example, sequence ATCGAT turned into pieces like AT, TC, CG, GA, and AT when K=2. These chunks serve as DNA fingerprints for various applications, such as database creation or genome assembly. The choice of K affects the trade-off between simplicity and complexity in the chunks. A smaller K yields more fragments that are simpler but may lack detail. A larger K gives fewer, more complex fragments that encapsulate more information about the original sequence.

#### Triplet encoding

Triplet encoding focuses on encoding DNA sequences using triplets of codons based on the genetic code’s 64 possible codons. Unlike K-mer encoding, where the value of K can vary, triplet encoding fixes K at 3, so that the sequence is always read three nucleotides at a time. For example, a DNA sequence like ATCGAT would yield non-overlapping triplets ATC and CGA. Each of the 64 possible codon triplets is assigned a unique identifier. These identifiers are then organized in a specific order, such as alphabetical or numerical order^52^. A repeat function then maps each nucleotide to its corresponding triplet identifier. However, triplet encoding also has limitations. For instance, the method relies on a predetermined list of 64 triplets, which may not be comprehensive enough to cover all possible DNA sequences. Moreover, it is not highly robust to errors or mutations. Even minor changes in the DNA sequence can significantly alter the resulting triplet identifiers, making the encoding less reliable compared to other schemes.

#### K-mer natural vectors

K-mer natural vectors represent a DNA or RNA sequence that quantifies the composition of k-mers (short contiguous substrings of length k) in the sequence. It is designed to overcome the deficiencies of previous k-mer models and provide a one-to-one mapping between a virus genome and its k-mer natural vector. This representation encodes the sequence into a high-dimensional vector, where each dimension corresponds to the frequency of each k-mer in the sequence^14^. Therefore, while K-mer encoding is simple but limited in its applications, K-mer natural vector provides greater versatility.

#### Voss representation

Voss representation encodes gene sequences using a series of binary strings^53^. Developed by Jeffrey Voss in the 1990s, it is a way to represent genomic sequence complexity using a simple binary code. A DNA sequence must first be broken up into individual nucleotides to be represented with the Voss representation. Following that, each nucleotide is assigned to the corresponding binary string. Concatenating the resulting binary strings creates a single, lengthy binary string that represents the whole DNA sequence. The Voss representation approach to encoding gene sequences has the benefit of being straightforward and user-friendly. Compact and effective binary codes can express lengthy and complex DNA sequences. It has certain drawbacks, though, namely its sensitivity to errors and mutations in the DNA sequence.

### Distance metrics

The similarity between encoded and non-encoded procedures is measured by distance metrics. A small difference between the encoded and non-encoded techniques suggests that they are similar, whereas a significant difference suggests that the encoded method does not capture the necessary characteristics of the non-encoded approach.

#### Euclidean distance

A genetic sequence can be thought of as a multidimensional vector where a different position in the sequence is represented by a unique dimension. The following equation is used to compute the Euclidean distance

3 $$\begin{aligned} d(a,b)= \sqrt{\sum \left(a[i]-b[i]\right)^2}. \end{aligned}$$

The equation calculates the square of the difference between each position in the two sequences. It sums these values, and then takes the square root of the result to get the final distance value. The Euclidean distance^54^ is a popular metric for measuring evolutionary relationships and has been widely used in similar domains^55,56^.

#### Tiples metric

The triple metric^57^ is a way to evaluate the accuracy of a phylogenetic tree in reconstructing evolutionary relationships between a set of taxa. A phylogenetic tree depicts the evolutionary history of a taxa and phylogentic inferences uses molecular or morphological data to reconstruct the branching pattern of such a tree.. The triplets metric evaluates the ability of a phylogenetic tree to correctly group three taxa together in a branching pattern based on their pairwise distances^58^.

#### Robinson-Foulds (RF) metric

The Robinson-Foulds^59,60^ metric is a popular method for comparing topological differences between two phylogenetic trees. The RF distance is based on the number of bipartitions present in one tree but not in the other. A bipartion divides a set of taxas into two groups such that are the taxa in one group are more similar to each other than any other taxas of the other group. The RF distance between tree A and tree B is the sum of bipartions present in tree A and not tree B, divided by two. Division by two is necessary to ensure RF distance is a metric. This means that it satisfies symmetry, non-negativity, and triangle inequality.

#### Matching pair metric

A matching pair metric^61^ is used to calculate the distance or similarity between two paired taxa in a phylogenetic tree. It can also be used to compare evolutionary relationships between two closely related taxas and to identify tree regions where different taxa groups are more related to each other.

#### Nodal splitted weighted distance metric

In Nodal splitted weighted^62^, the tree topology and branch lengths are estimated by minimizing the sum of weighted distances between the observed sequences and the reconstructed sequences at internal nodes of the tree. Each node’s weight is dependant on the number of sequences contained in it. The least squares method is employed to estimate branch lengths. In addition to be computionally efficient and scalable to massive datasets, it offers more accuracy than other distance based methods when number of sequences is large or with sequences with considerable evolutionary distances between them.

#### Matching cluster distance

Matching cluster distance^61^ is a distance metric used in phylogenetic tree construction. MCD is based on pairwise distances between clusters of sequences rather than pairwise distances between individual sequences.

#### MAST

Maximum Agreement Subtree^63^, or known as MAST is a distance metric used to compare phylogenetic trees, specifically the similarity between two trees. This metric measures the distance between two trees based on the size of their maximum agreement subtrees. A maximum agreement subtree is a subtree that appears in both trees and has the maximum number of nodes. The MAST distance is the difference between the total number of nodes in the two trees and twice the size of their maximum agreement subtree. In other words, it measures the number of nodes pruned from one tree to obtain the other.

#### Cophenetic L2

Cophenetic L2^64^ distance is a metric used to compare the cophenetic distance matrices of two trees.

4 $$\begin{aligned} d = \sqrt{\sum \left(A[i][j] - B[i][j]\right) ^2}, \end{aligned}$$

where, A[i][j] and B[i][j] represents the points at the i-th row and j-th column, respectively. It is primarily used to assess the quality of different tree reconstruction methods.

#### Quartet distance

The quartet distance^65^ metric is used in unrooted phylogenetic tree reconstruction to evaluate the similarity or dissimilarity between different trees. It is a distance metric that measures the difference between two trees based on the number of quartet trees they share. A quartet tree has four taxas and the branching pattern joins two taxa pairs. For instance a quartet tree with taxa W, X, Y, Z could have ((W,X), (Y,Z)) or ((W,Y), (X,Z)) branching patterns.. The quartet distance metric calculates the number of quartet trees shared between two trees.

#### Path difference

The path difference^66^ metric assesses the robustness of branches in a phylogenetic tree. This is done by calculating the path difference between the original tree and a reference tree with a particular branch removed. If the path difference is small, the branch is considered well-supported and robust, while a larger path difference indicates a less robust branch.

## Results and discussion

The encoded methods are put to the test using various datasets belonging to viruses like SARS-CoV-2 and influenza, which include both short and long genomes. To evaluate and compare the different genomic data, moment vectors were computed on each method. These vectors represent the statistical properties of the sequence data and are used to compare the similarity between different sequences. A matrix was created showing the pairwise distances between these vectors, which was used to cluster the data into biological groups to construct phylogenetic trees. Further process involves comparing the encoded and non-encoded methods to determine their similarities. This is achieved by creating a normalised matrix for each method and using the Euclidean distance for comparison. Accordingly, this comparison enables understanding of how the encoded and non-encoded methods relate to each other and identifies any similarities between them.

**Figure 2:**
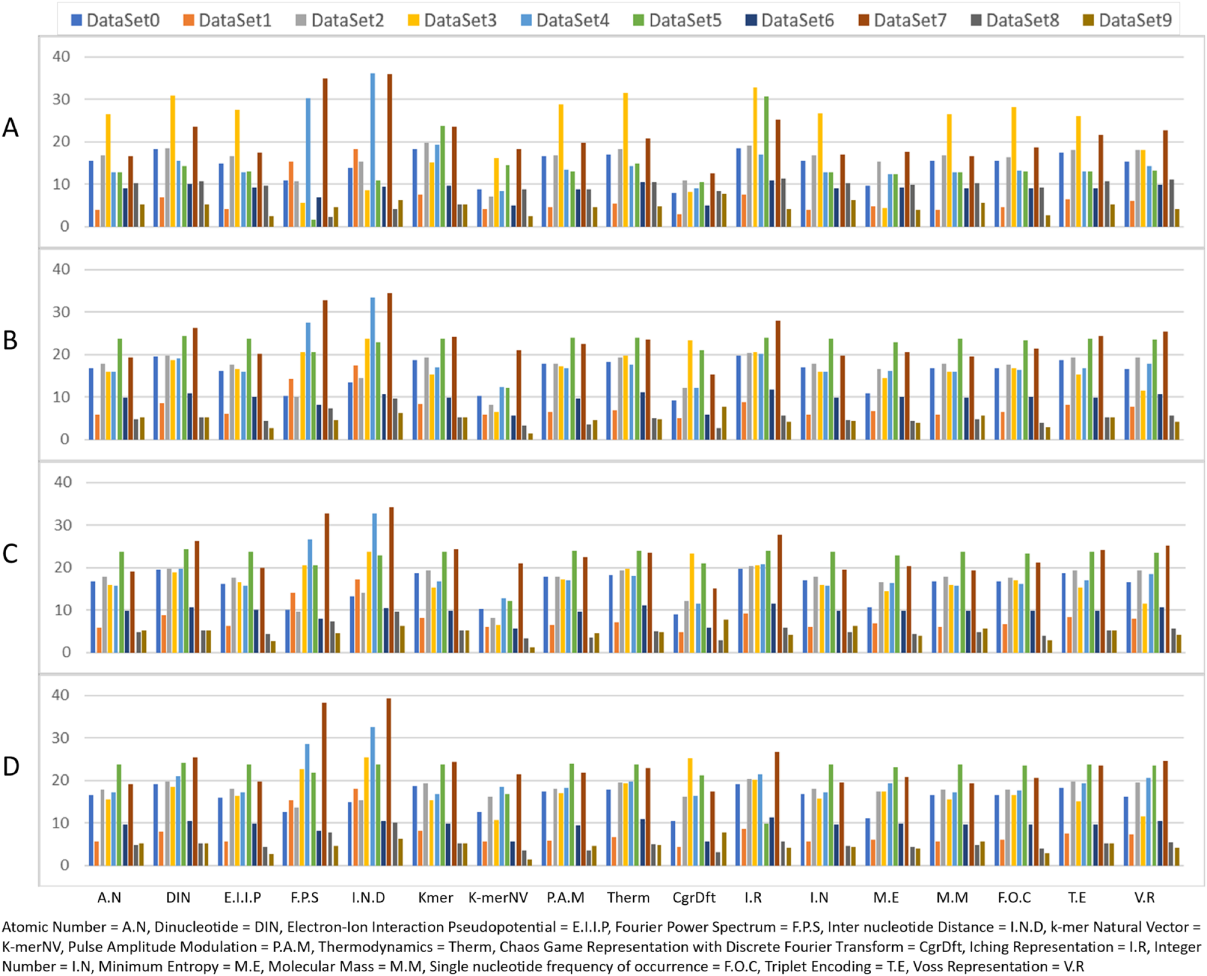
A comparison of Euclidean distances among distance matrices generated by encoding and non encoding techniques across ten distinct datasets (DataSet 0-9). Each bar represents the euclidean distance between distance matrices generated by encoded method (X-axis) and non-encoded multi-sequence alignment (**A**) ClustalW (**B**) ClustalOmega (**C**) MAAFT (**D**) MUSCLE methods.

Figure 2 represents the Euclidean distance values for various encoding methods in comparison to non-encoded multi-sequence alignments generated by four different methods: ClustalW, ClustalOmega, MAAFT, and MUSCLE for ten different datasets (DataSet 0–9) where each bar represents the Euclidean distance between the distance matrices of the respective encoded and non-encoded method for a particular dataset.The minimum euclidean distance value for each dataset highlights the method that is most similar to the non-encoded method for that dataset. For each dataset, Chaos Game Representation with Discrete Fourier spectrum (CgrDft), K-mer natural vectors (K-merNV), Fourier Power Spectrum(FPS) were the encoding techniques that had the least Euclidean distances with non-encoded techniques used for comparison in this paper.

Further analysis on the performance of encoded method shows more than 80% of the time, CgrDft and K-merNV methods have similarity with popular non-encoded methods ClustalW (Fig. 2A),ClustalOmega (Fig. 2B),MAAFT (Fig. 2C) and MUSCLE (Fig. 2D). This indicates that for these datasets, the CgrDft and K-merNV methods are the most effective encoding techniques among the methods listed. In other words, the CgrDft and K-merNV encoding methods have the highest similarity to the non-encoded methods, suggesting that they produce the most accurate results for these datasets. Thus, the CgrDft and K-merNV encoding techniques could be a better choice for encoding sequences when performing sequence analysis on these datasets.

**Figure 3:**
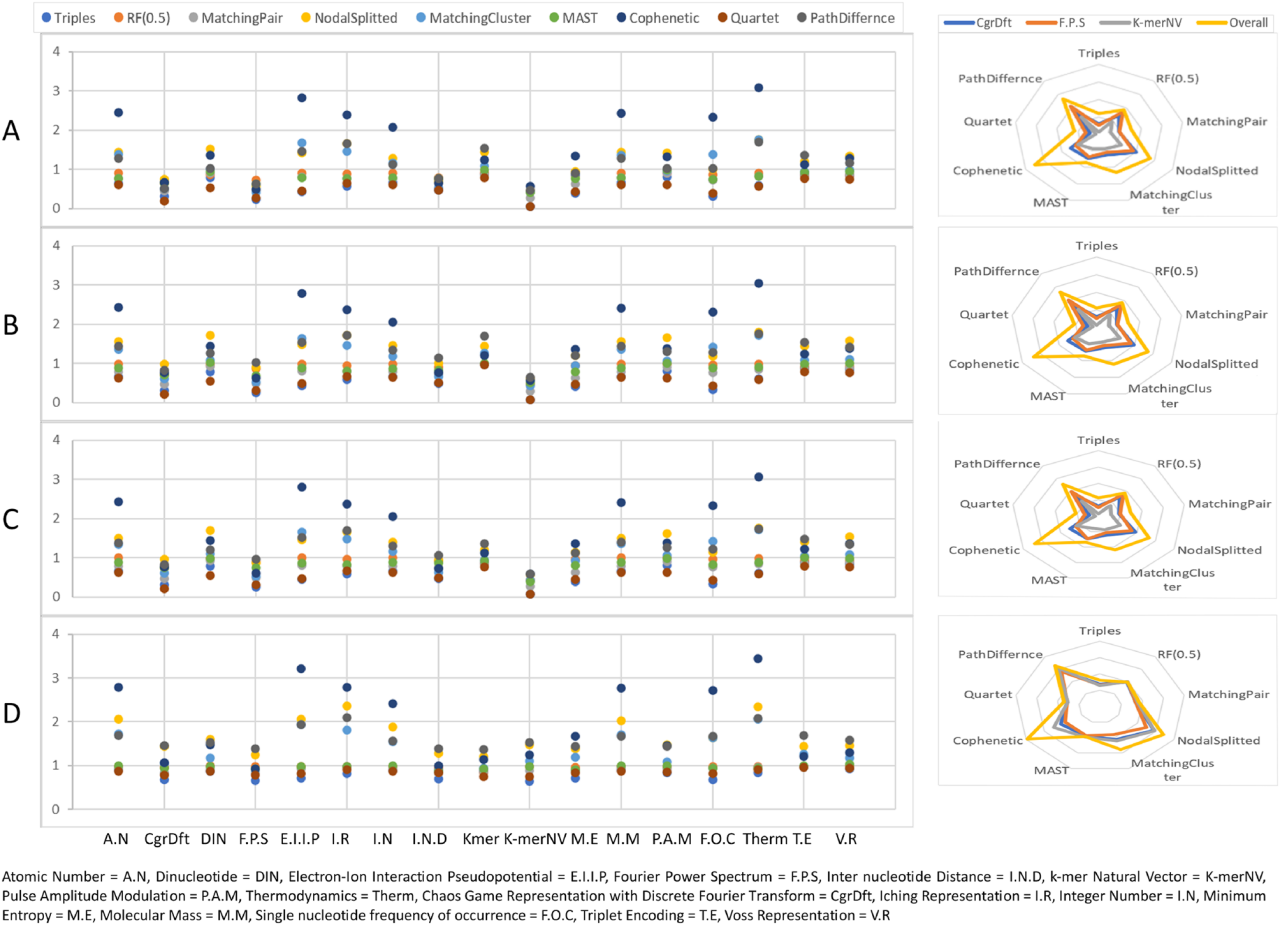
A representation of metrics (Triples, RF(0.5) etc) applied to phylogenetic trees generated by encoding and non-encoding techniques across the dataset (DataSet 0). Each dot represents the metric value between phylogenetic trees generated by encoded method (X-axis) and non-encoded multi-sequence alignment (**A**) ClustalW (**B**) ClustalOmega (**C**) MAAFT (**D**) MUSCLE methods. Radar plot shows the position of distance metrics Triples, RF(0.5), MatchingPair, NodalSplitted, MatchingCluster, MAST, Cophenetic, Quartet, and PathDiffernce) for K-merNV, CgrDft, and FPS.

To further validate these results, we applied the visual TreeCmp^67^ package for distance metrics tests on arbitrary non-binary phylogenetic trees generated by each encoded method. Here, the reference trees to which each encoded method tree is compared are generated by non-encoded methods(ClustalW, ClustalOmega, MAAFT and MUSCLE). The marked points in Fig. 3 indicate that the values for all mentioned metrics are lower for CgrDft, K-merNV, and FPS encoding methods. Furthermore , Fig. 3 shows the radar chart for values for these methods and the overall average values of all methods. The overall average value is obtained by comparing the 68 values (i.e., 17 × 4) from all encoded techniques (a total of seventeen methods) to all non-encoded methods (a total of four methods). As it can be seen, the position of each distance metric is under the overall values for CgrDft, K-merNV, and FPS encoding methods which validates the results from Euclidean distance. To further validate these findings, methods with the lowest values, such as CgrDft, K-merNV, and FPS were visually compared.

Figures (Supplementary Figs. 1–4) depict phylogenetic trees (based on Dataset0) demonstrating the evolutionary relationships between different virus strains based on their genetic sequences. These trees are created using shortlisted encoded methods from the previous step i.e., K-merNV, CgrDft, FPS, and clustalW. The supplementary material contains phylogenetic tree files that include all methods, both encoded and non-encoded, across all datasets, which can be used to visually compare the performance of different methods.

These phylogenetic trees are a detailed and complete representation of the evolutionary relationships among viruses, including SARS-CoV-2, which is currently the most pathogenic coronavirus strain. It displays a larger number of branches, providing more information about the relationships between strains. The lengths of the branches on the tree show how much evolution has occurred since the divergence from a common ancestor. The overall pattern of branching gives meaningful insights into relationships. A short branch does not necessarily signify a lesser connection, nor does a long one indicate a more substantial relationship. It is the way the branches connect that counts.

Comparing the different trees reveals that the K-merNV (Supplementary Fig. 2) and multi-sequence alignment methods (Supplementary Fig. 1) produce consistent and accurate results, reflecting the similar grouping between the virus sequences. In contrast, CgrDft (Supplementary Fig. 3) and FPS (Supplementary Fig. 4) methods show incorrect grouping of some strains. Specifically, CgrDft incorrectly grouped ’Rousettus bat CoV HKU9,’ while FPS incorrectly grouped ’TGEV’, ‘Bat CoV RaTG13’ and ’Mink CoV WD1127’. Therefore, the K-merNV phylogenetic tree appears to be the most comprehensive and accurate representation of multi-sequence alignment methods, followed by CgrDft and FPS.

While our study has shown promising results in understanding the genetic relationships among viruses, it mainly looked at a subset of viruses. However, these methods can also be applied to other viruses. For example, recent research has used alignment-free techniques to study phages which have diverse genetic material^68,69^. Moreover, our focus was on some particular techniques for analysing genetic sequences, however, there are newer methods^70–75^ that use advanced computer algorithms to possibly get even more accurate results.

## Conclusion

This study highlights the potential of encoded methods for classifying viruses and phylogenetics. While previous studies only compared the effectiveness of each method by comparing their phylogenetic trees, this paper compares the vectors generated by encoded and non-encoded methods using distance metrics to determine similarity between them. Through these comparisons, we evaluate how well the encoded methods perform in comparison to existing, widely used alignment methods. By comparing the results of the encoded methods to those of ClustalW and MUSCLE (implemented on MEGA 11) and MAFFT and ClustalOmega (implemented on NGphylogeny), we determine the K-merNV followed by CgrDft encoded methods are similar with the current state of the art-multi sequence alignment methods. To the best of our knowledge, this is a novel approach to incorporation and comparison for encoded methods. In the future, examining the behaviour of encoded methods when tested on other distance vectors, such as those generated using Kimura and Tamura models, might be of interest. Further, it would be interesting to see some advanced algorithms to improve these encoded methods. Another interesting avenue could be to compare alignment-based methods with the latest alignment-free methods to see which ones provide the most accurate results. It would also be insightful to explore the use of encoded methods in other areas of genomics research and compare their performance to existing methods in those domains. Furthermore, making these methods faster and more accurate could be a game-changer for getting important information more quickly.

### Supplementary Information

Supplementary Figures.

## Data Availability

All data generated or analyzed during this study is available on GitHub, https://github.com/marslanshaukat/Encoded-and-Alignment-Based-Methods.git.
