# Supplementary material for: Comparative study of encoded and alignment-based methods for virus taxonomy classification
Source: Sci Rep. 2023 Oct 31;13:18662. doi: 10.1038/s41598-023-45461-0 (PMC10618506; doi:10.1038/s41598-023-45461-0)
Supplement: Supplementary file 1 — Supplementary Figures. [file 41598_2023_45461_MOESM1_ESM.pdf]

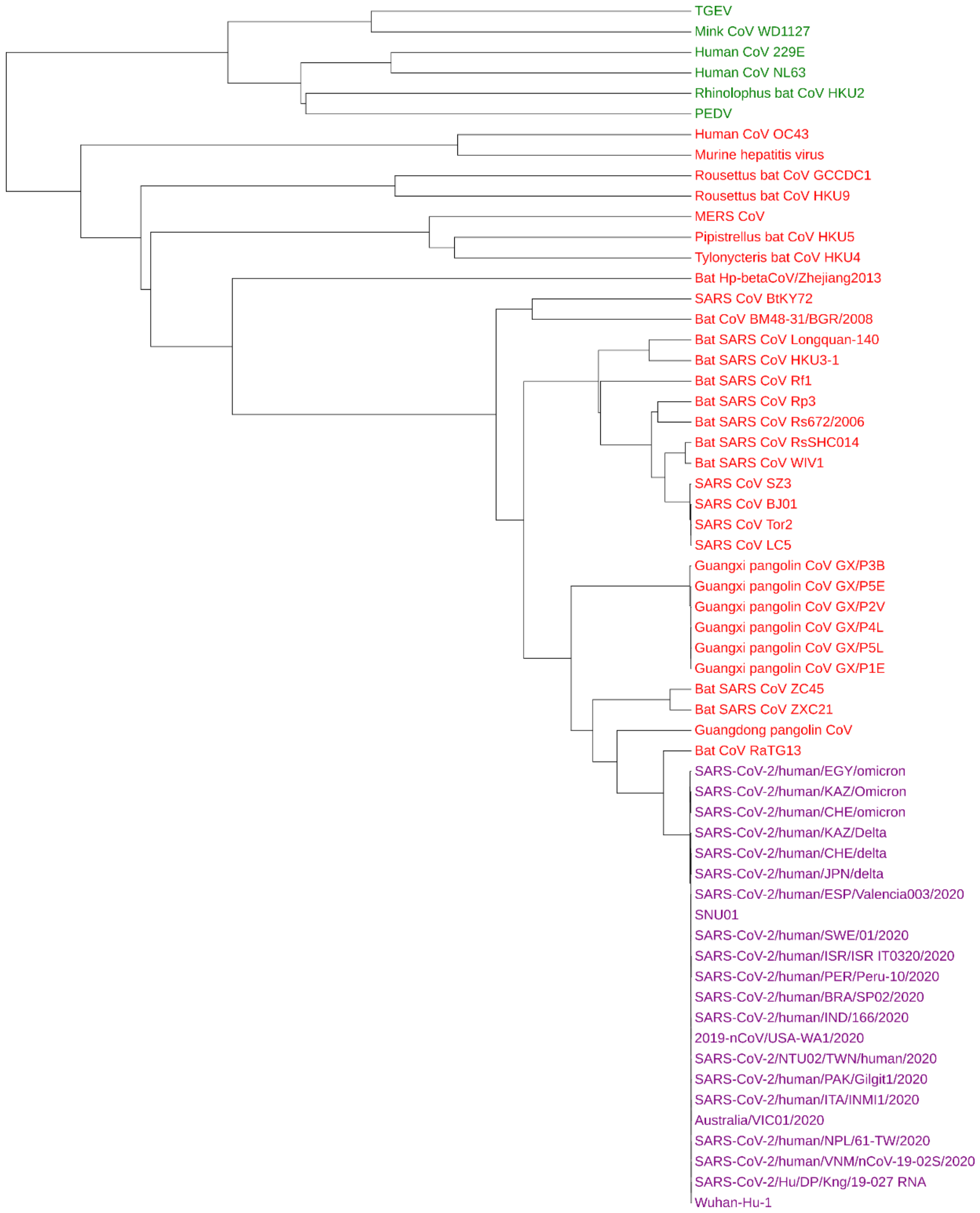

Supplementary Figure 1. A phylogenetic tree using Dataset0 for ClustalW method for viruses in the genus AlphaCoV(green), BetaCoV(red), and 22 representative SARS-CoV-2 sequences(purple).

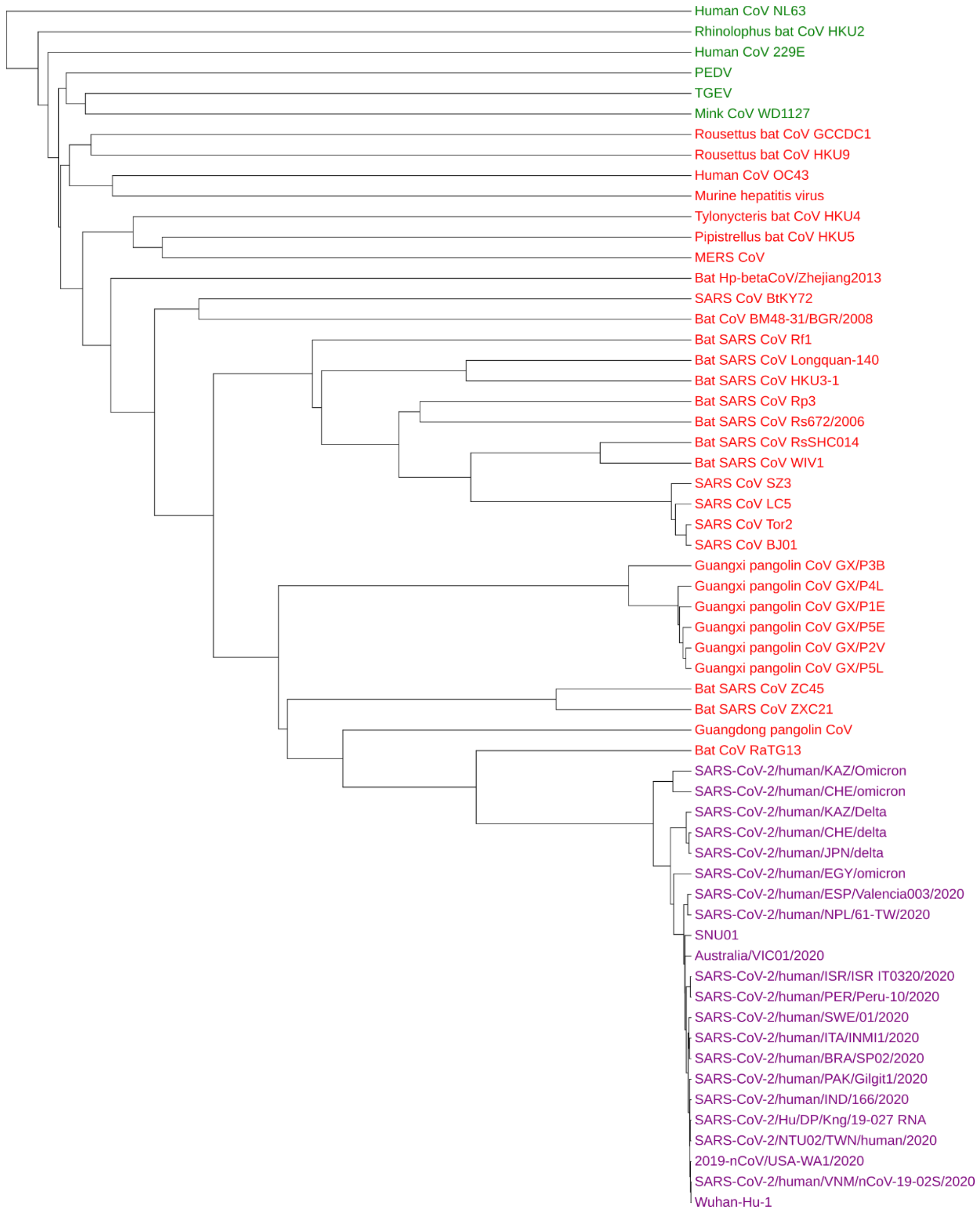

Supplementary Figure 2. Phylogenetic tree created using Dataset0 for K-merNV. The results indicate that K-merNV and ClustalW produced similar groupings.

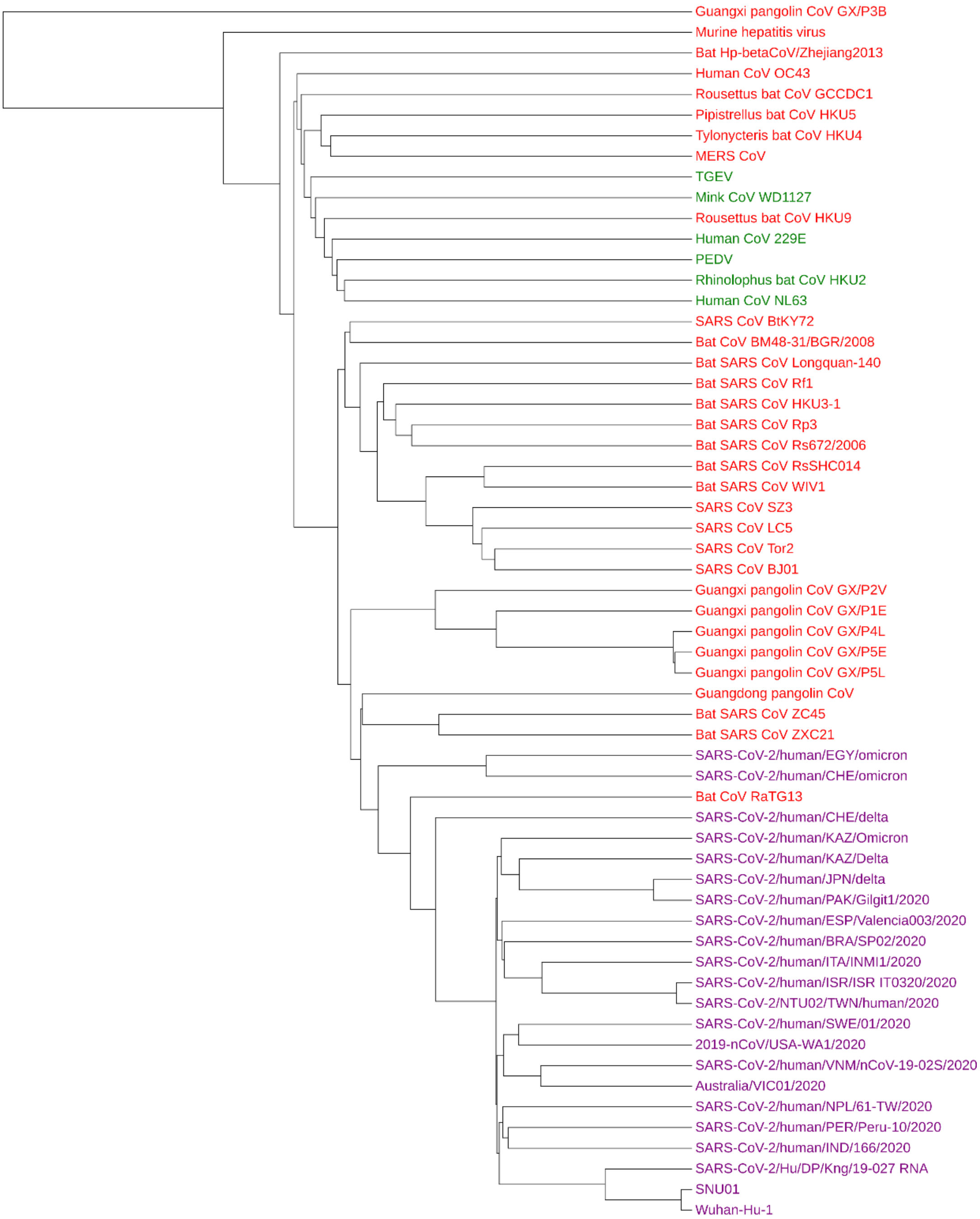

Supplementary Figure 3. Phylogenetic tree created using Dataset0 for CgrDft methods. The results indicate that CgrDft incorrectly grouped multiple sequences when compared with ClustalW.

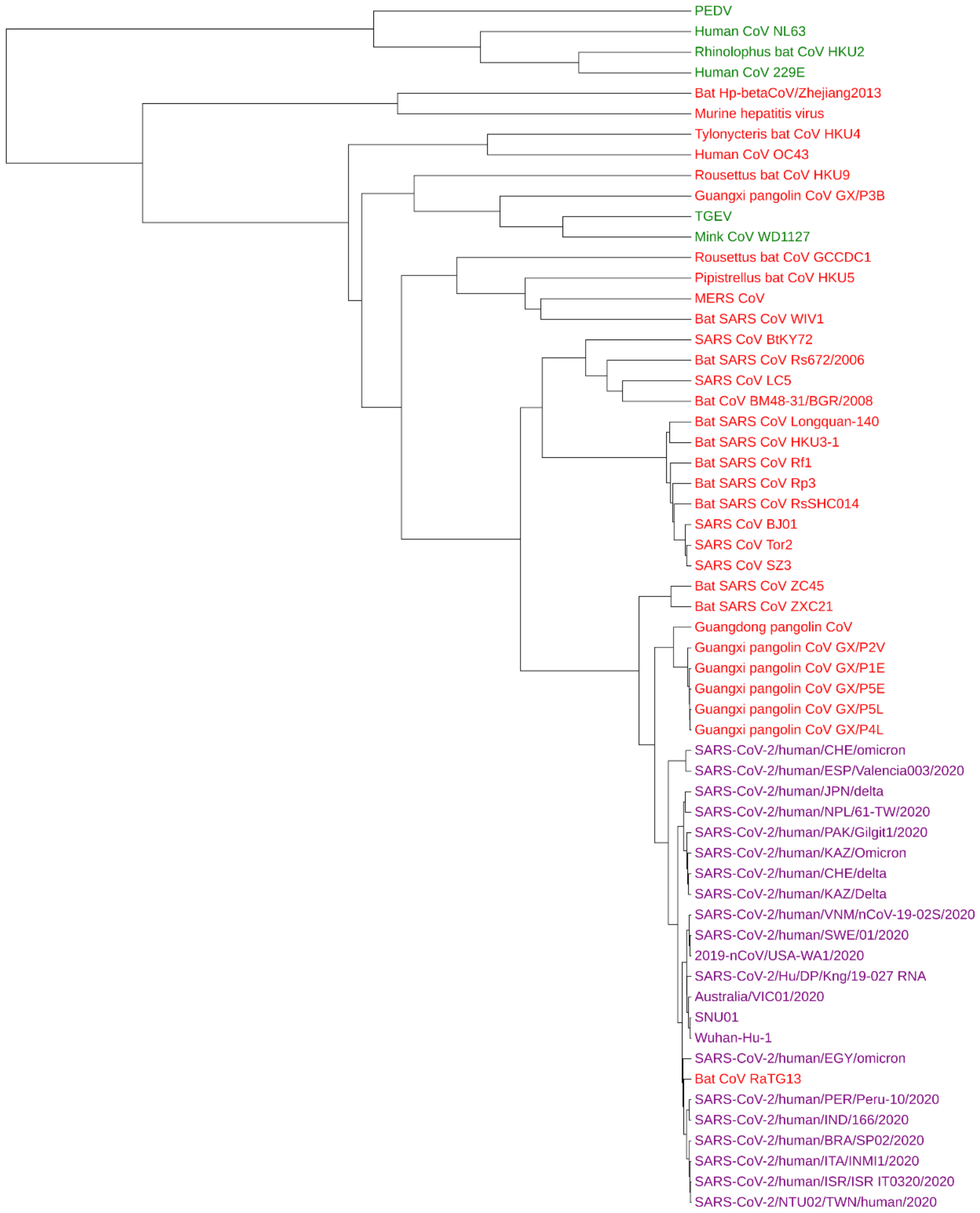

Supplementary Figure 4. Phylogenetic tree created using Dataset0 for FPS method. The results indicate that FPS incorrectly grouped more than two sequences when compared with ClustalW.
